# Small Molecule-BIO Accelerates and Enhances Marrow-Derived Mesenchymal Stem Cell in Vitro Chondrogenesis

**Published:** 2014-03

**Authors:** Mohamadreza Baghaban Eslaminejad, Nasrin Fallah

**Affiliations:** 1 Department of Stem Cell and Developmental Biology at Cell Science Research Center, Royan Institute for Stem Cell Biology and Technology, ACECR, Tehran, Iran;; 2 Department of Developmental Biology, University of Science and Culture, Tehran, Iran

**Keywords:** Mesenchymal stem cells, Mouse, 6-bromoindirubin-3-oxim

## Abstract

**Background: **Hyaline cartilage defects exhibit a major challenge in the field of orthopedic surgery owing to its limited repair capacity. On the other hand, mesenchymal stem cells (MSCs) are regarded as potent cells with a property of cartilage regeneration. We aimed to optimize marrow-derived MSC chondrogenic culture using a small bioactive molecule referred to as BIO.

**Methods: **MSCs from the marrow of NMRI mice were extracted, culture-expanded, and characterized. Micro-mass culture was then established for chondrogenic differentiation (control group). The cultures of MSC in chondrogenic medium supplemented with 0.01, 0.05, 0.1, and 1 µM BIO were taken as the experimental groups. Cartilage differentiation was examined by both histological sections and real-time PCR for Sox9, aggrecan, and collagen II at different time points. Moreover, the involvement of the Wnt pathway was investigated.

**Results:** Based on histological sections, there was seemingly more intense metachromatic matrix produced in the cultures with 0.01 µM BIO. In this experimental group, cartilage-specific genes tended to be upregulated at day 14 compared to day 21 of the control group, indicating the accelerating effect of BIO on cartilage differentiation. Overall, there was statistically a significant increase (P=0.01) in the expression level of cartilage-specific genes in cultures with 0.01 µM BIO (enhancing effects). These upregulations appeared to be mediated through the Wnt pathway evident from the significant upregulation of T-cell factor and beta-catenin molecules (P=0.01).

**Conclusion: **Taken together, BIO at 0.01 µM could accelerate and enhance in vitro chondrogenesis of mouse marrow-derived MSCs.

## Introduction

The treatment of injuries to the hyaline cartilage is considered a challenge in the field of orthopedic surgery. This is because of very limited repair capacity of the hyaline cartilage. Chondrocytes in the mature cartilage have lost their ability to undergo proliferation and are, hence, unable to participate in the repair process. Furthermore, the cartilage is described as an avascular tissue. The existence of blood vessels is necessary for triggering an inflammatory response, which brings repair cells, including monocytes and macrophages, to the injury site. Often hyaline cartilage defects fill with fibrocartilage, which is not biomechanically suitable for weight-bearing.^1^^,^^2^ Current therapies used in the clinic to reconstruct the cartilage tissue include marrow stimulation techniques such as microfracture, osteochondral mosaicplasty, and cell-based treatments.^3^^-^^5^

There are two types of cell-based treatments for cartilage defects: autologous chondrocyte implantation (ACI) and mesenchymal stem cell (MSC)-based therapy.^6^ ACI involves the preparation of chondrocytes from an intact region of the cartilage and their culture-expansion and transplantation by surgery. This technique involves a two-step surgical procedure: one for collecting the tissue and the other for the transplantation of the cells. Moreover, obtaining a sufficient number of chondrocytes from the tissue biopsies is challenging; therefore, in vitro expansion of the cells is inevitable. It has been reported that chondrocytes expanded in culture gradually undergo dedifferentiation and loose morphological features as well as specialized functions.^7^

Considering the drawbacks associated with chondrocytes and in the search for better cell source, MSCs have been found a suitable candidate for application in cartilage regeneration thanks to their extensive self-renewal property and chondrogenic differentiation capacity.^8^^,^^9^ MSCs were first described by Fridenstein et al.^10^^,^^11^ from bone marrow tissue as colonogenic fibroblastic cells capable of producing bone and cartilage-like tissues in culture. There are two fundamental parameters that must be provided in culture in order to induce MSCs towards cartilage differentiation; these include close interaction between the cells and the presence of chondrogenic inducers in the culture medium.^12^^-^^16^

In cell-based treatment of tissue defects, one strategy is to transplant fully-differentiated cells into the injured site. For this reason, the subject of the optimization of MSC chondrogenic differentiation is of particular importance.^17^ Some investigations have indicated that the differentiation of MSCs into cartilage cells occurs following the activation of certain signaling pathways, particularly the Wnt (wingless type) pathway. One key molecular regulator of the Wnt pathway is the glycogen synthase kinase-3 (GSK-3) enzyme. The inhibition of this molecule initiates the signaling pathway.^18^^-^^20^ On the other hand, some investigators have reported that a small molecule referred to as BIO (6-bromoindirubin-3–oxim), derived from Tyrian purple indirubins, possesses a GSK-3-selective inhibitory function. It acts by binding on a groove between ATP and GSK-3ß, resulting in the activation of the Wnt signaling pathway.^21^

A number of investigations have so far been conducted using the Wnt-activating property of BIO. These studies have reported some interesting effects of this small molecule. Some have found that the addition of BIO into the cell culture medium results in culture protection against apoptotic changes.^22^^,^^23^ Others have concluded that the presence of BIO in the culture medium enhances the growth capacity of the cultured cells.^24^^,^^25^ Finally, a few studies have reported that BIO supplementation leads to the maintenance of pluripotency in embryonic stem cell culture.^26^^-^^28^

There is no report regarding the effect of BIO on MSC in vitro chondrogenesis. The objective of the present investigation was to examine whether or not the addition of BIO into the culture medium could improve cartilage differentiation of marrow-derived MSCs.

## Materials and Methods

*Animals*

MSCs from 10 NMRI male mice (4-8 weeks old) were studied in the current experimental study. Prior to the experiment, approval for animal use was obtained from the Ethics Committee of Royan Institute. 

*Bone Marrow Cell Culture*

The mice were killed by cervical dislocation, and their tibia and femur were removed and transferred to the cell culture lab. Within laminar cabinet, the bone marrow was flushed out of the medullary canal using an insulin needle inserted into the clipped end of the long bones. The marrow was mixed with 5 ml DMEM (Dulbecco’s Modified Eagle Medium, Gibco, Germany) containing 15% FBS (fetal bovine serum, Gibco, Germany) and 100 IU penicillin and 100 µg/ml streptomycin (Gibco, Germany) and centrifuged at 400 g for 3 min. The cell pellet was suspended in DMEM, cultivated at 10^ 6 ^-cells/ml in 75-cm culture flasks, and incubated in an atmosphere of 5% CO2 at 37ºC. The medium was replaced twice weekly until the culture reached confluency. At this time, the cultures were subcultured with a 1:3 ratio into new culture flasks. Passaged-3 cells were used at the following experiments.

*Flow Cytometry of Cell Surface Markers*

To ensure that the isolated cells were of the MSC population, the expression of some known mesenchymal surface markers, including CD73 and CD44, and the absence of hematopoietic (CD34, CD11b) and endothelial cell markers (CD31) were investigated using flow cytometric analysis. In brief, 10^ 6^ passaged-3 cells were placed in 5-ml tubes and added with 5 µl of each antibody and 5 µl of blocking buffer. The cells were incubated in the dark at 4°C for 20-25 min, washed with PBS supplemented with 1% FBS and centrifuged at 400 g for 2 min. The cell pellet was then suspended in 300-500 µl washing buffer and analyzed by flow cytometry (FACScalibur cytometer equipped with 488 nm argon lasers). In this study, IGG2 and IGG1 were used as isotope control. WinMDI software was used to analyze the flow cytometric results.

*Multilineage Differentiation*

Since the golden criterion for the identification of a cell population as MSC is the differentiation assay, the isolated cells were examined to find whether or not they were able to give rise to osteocytic and adipocytic cell lineages. For this purpose, a confluent culture of the passaged-3 cells was established and treated with either osteogenic medium consisting of DMEM supplemented with 50 mg/ml ascorbic 2-phosphate (Sigma, USA), 10 nM Dexamethasone (Sigma, USA), and 10 mM β glycerol phosphate (Sigma, USA) or adipogenic medium consisting of DMEM supplemented with 50 μg/ml ascorbic acid 3-phosphate, 100 nM Dexamethasone, and 50 μg/ml Indomethacin. The cultures were incubated at 37ºC and 5% CO2 for 3 weeks. At the end of this period, the cultures were fixed and stained by either Alizarin Red for bone mineralized matrix or Oil Red for cytoplasmic lipid droplets. The differentiations were also checked by RT-PCR for the specific bone markers, including osteocalcin and Runx2, and adipose, including PPAR gamma (peroxisome proliferators-activated receptor gamma) and LPL (Lipoprotein lipase).

*RT-PCR Analysis*

Total RNA was isolated from the osteogenic and adipogenic cultures using Trizol (Invitrogen). The RNA sample was treated with 1 U/µl of RNase-free DNaseI (EN0521; Fermentas, Opelstrasse 9,Germany) per 1 µg of RNA in the presence of 40 U/µl ribonuclease inhibitor (E00311; Fermentasm, Germany) and 10x reaction buffer with MgCl2 for 30 min at 37°C to eliminate residual DNA. DNaseI was inactivated by adding 1-2 µl of 25 mM EDTA and incubated at 65°C for 10 min. Standard RT reactions were performed with 2 μg total RNA using random hexamer as a primer and a RevertAid^TM^ First Strand cDNA Synthesis Kit (Fermentas, Germany) according to the manufacturer’s instructions. For every reaction set, one RNA sample was prepared without RevertAid MMuLV Reverse Transcriptase (RT-reaction) in order to provide a negative control of the subsequent PCR. To minimize variation in the RT reaction, all the RNA samples from a single experimental setup were simultaneously reverse-transcribed. Reaction mixtures for PCR included 2 µl cDNA, 10xPCR buffer (AMSTM; CinnaGen Co., Tehran, Iran), 200 mM dNTPs, 1.5-2 mM MgCl2 (CinnaGen Inc., Tehran, Iran), 0.5 mM of each antisense and sense primer, and distilled water to reach to the total volume. The RT-PCR reaction was performed in 25 µl.

*Chondrogenic Cultures and BIO Treatment*

To establish chondrogenic culture, a micromass culture system was used. Also, the 2.5×10^ 5^ passaged-3 cells was pelleted under 400 g for 5 min and provided with chondrogenic medium made of DMEM supplemented with 10 ng/ml TGF- ß3 (transforming growth factorß3) (Sigma, Germany), 10 ng/ml BMP6 (bone morphogenetic protein-6) (Sigma, Germany), 50 mg/ml insulin transferrin selenium+premix (Sigma, Germany), 1.25 mg bovine serum albumin (Sigma, Germany), and 1% FBS for 3 weeks.

To investigate the effect of BIO (Sigma-Aldrich, Germany) on MSC in vitro chondrogenesis, the chondrogenic medium was supplemented with 0.01, 0.05, 0.1, and 1 µM concentrations of BIO, which were prepared using DMSO (Dimethyl Sulfoxide, Sigma-Aldrich, Germany) as a solvent. The culture without BIO and containing the same volume of DMSO as the BIO groups was taken as the control. All the cultures were incubated in an atmosphere of 5% CO2 at 37ºC for 21 days. At the end of this period, some pellets were prepared for light microscopic studies and the others were used for RT PCR, which was carried out at different time points, including days 5, 14, and 21. With quantitative PCR, two sets of molecular markers were analyzed: cartilage-specific genes, including Sox9, aggrecan, and collagen II and the Wnt signaling pathway-related key molecules such as TCF (T-cell factor) and beta-catenin.

*Light Microscopy*

The chondrogenic pellets were fixed overnight in 10% formaldehyde in PBS buffer, washed with tap water, incubated in an ascending row of isopropanol (Merck, Darmstadt, Germany), cleared in xylene, and finally embedded in paraffin wax (Leica, Bensheim, Germany). The blocks were then cut into 5-μm-thick sections, which were stained with Toluidine blue. 

*Quantitative Real-Time RT-PCR*

To quantify relative gene expression levels, total RNA was extracted from the cell samples using Trizol (Invitrogen). cDNA was synthesized from total RNA using a RevertAid^TM^ First Strand cDNA Synthesis Kit (Fermentas, Germany) according to the manufacturer’s instructions. Aggrecan, collagen II, and Sox9 mRNA levels, as chondrogenic differentiation marker genes, were measured by RT-PCR (Stepone RT PCR Applied BIOsystems, USA). The 20 μL reaction contained 2 μL cDNA from each sample mixed with 10 μL SYBR® Green PCR Mastermix (Invitrogen), 2 μL primers, and 6 μL RNase/DNase-free water. The PCR conditions were comprised of incubation at 95°C for 2 min, followed by 45 cycles at 95°C for 15 s and at 60°C for 60 s.

The gene expression levels of the target genes: collagen II, Sox9, and aggrecan were determined based on the threshold PCR cycle-values [Ct (target)] following the instructions of Applied BIOsystems. The relative quantification was derived using the Comparative CT method using 2^-ΔΔCt^, where the amount of the target is normalized to an endogenous control (beta actin) and relative to calibrator (samples without treatment). The specific primers designed for the target genes are listed in table 1.

**Table 1:** Primers used in RT-PCR

**Gene Name**	**Primer Sequence**	**Annealing Temperature**
Osteocalcin	Forward: GGC AAT AAG CTA GTG AAC AG Revers: GGT CCT AAA TAG TGA TAC CGT	60
Runx2	Forward: CAG CAT CCT ATC AGT TCC CAA Revers: CAG CGT CAA CAC CAT CAT	60
PPARgamma	Forward: GAG CAC TTC ACA AGA AAT TAC C Revers: AAT GCT GGA GAA ATC AAC TG	60
LPL	Forward :AAT TGT CCC ATG CTG TAA CC Revers:CAG GAC ACA GGA AGC TAA GG	60
CollagenII	Forward: GTTCACATACACTGCCCT Revers: GTCCACACCAAATTCCTC	60
Aggrecan	Forward: CCCAGAGAAATTCACTTCC Revers :TAGATAGACAGTCCTTACACCC	60
Sox9	Forward: CCAGCGTTTAACCTTCAAGAC Revers: ATTTAACAACAGATGACCATACCC	60
-cateninβ	Forward: AGAACACTAATTCATAATCACGCT Revers: GGCTCAAATAACACCTCTTACTG	60
TCF	Forward: TACCCTACAAATGCTTCTCCTG Revers: AAACGTATCCTAGTCCCTCCT	60
GAPDH	Forward: CAA CTC CCA CTC TTC CAC TT Revers: GCAGCGAACTTTATTGATGGTA	60

*Statistical Analysis*

All the measurements were performed in triplicate and the averages were analyzed and compared using the repeated measure ANOVA. P<0.05 was considered as statistically significant.

## Results

*Marrow Cell Culture*

**Figure 1:**
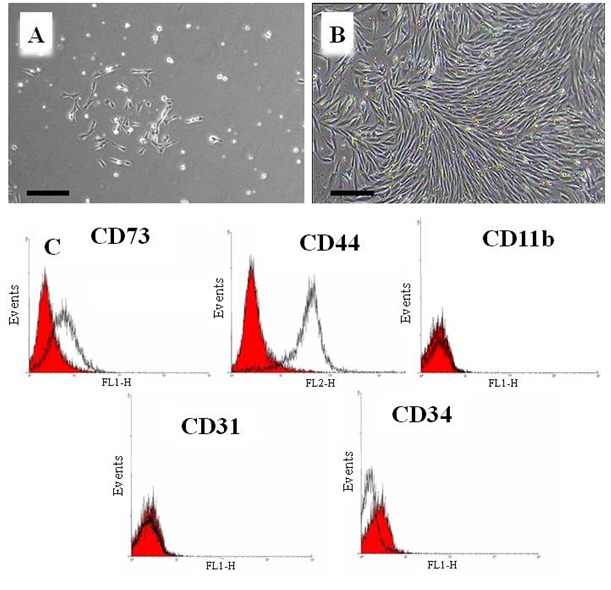
Mouse marrow-derived mesenchymal stem cells. Mouse marrow cells in primary culture before confluency at day 2 (A) and after confluency at day 10 (B), C) Majority of mouse marrow MSC expressed mesenchymal markers (CD73, CD44) and did not express hematopoietic (CD11b, CD34) and endothelial markers (CD31) (Bar=200 µm).

The primary culture of the marrow cells contained mostly fibroblastic cells along with a few small round cells (figure 1A). This culture became confluent in 10 days when the fibroblastic morphology dominated the culture (figure 1B). This morphology persisted throughout the cultivation period. At subcultures, the cells tended to rapidly proliferate reaching confluency in 7 days.

*Flow Cytometry*

While CD73 and CD44, two mesenchymal markers, were expressed by most cells, hematopoietic as well as endothelial cell markers such as CD31, CD11b, and CD34 were expressed at a very low percentage of the studied cells (figure 1C).

*Multilineage Differentiation Potential of MSCs*

**Figure 2:**
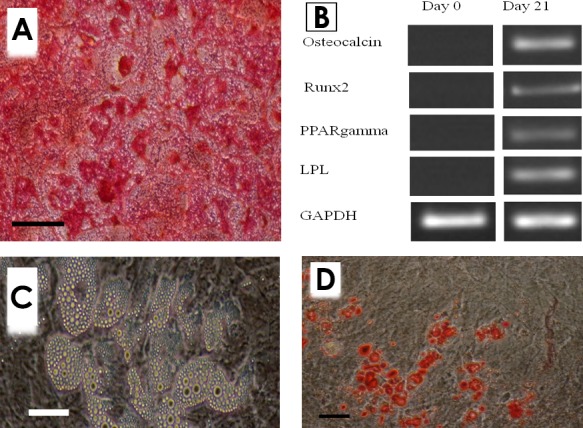
Multilineage differentiation potential of MSCs. A) Osteogenic culture of mouse marrow MSCs stained by alizarin red (Bar=200 µm), B) Unstained adipogenic culture of the same cells: lipid droplets in the fat cells are easily observable (Bar=50 µm), C) Adipogenic culture stained by Oil red (Bar=100 µm), C) Differentiated cells expressed bone and adipose-specific genes.

According to our observations, a number of cell aggregates were developed at osteogenic cultures a week after culture initiation. The number of the aggregates increased as the culture progressed. These osteogenic nodules tended to be positively stained with alizarin red, indicating the deposition of mineralized matrix in the culture (figure 2A). Based on the RT-PCR findings, the cultures tended to express bone-specific genes, including osteocalcin and Runx2 (figure 2B).

Small lipid-like droplets became visible at adipogenic culture at day 3 as the cells differentiated into adipose cells (figure 2C). The number of adipocytes was then progressively increased. Positive red staining of these droplets with Oil red indicated their lipid nature and confirmed the adipogenic differentiation of the studied cells (figure 2DD). The RT-PCR analysis indicated that the cells also expressed adipose-specific genes, including PPARgamma and LPL (figure 2B).

*Light Microscopy of Chondrogenic Culture*

**Figure 3:**
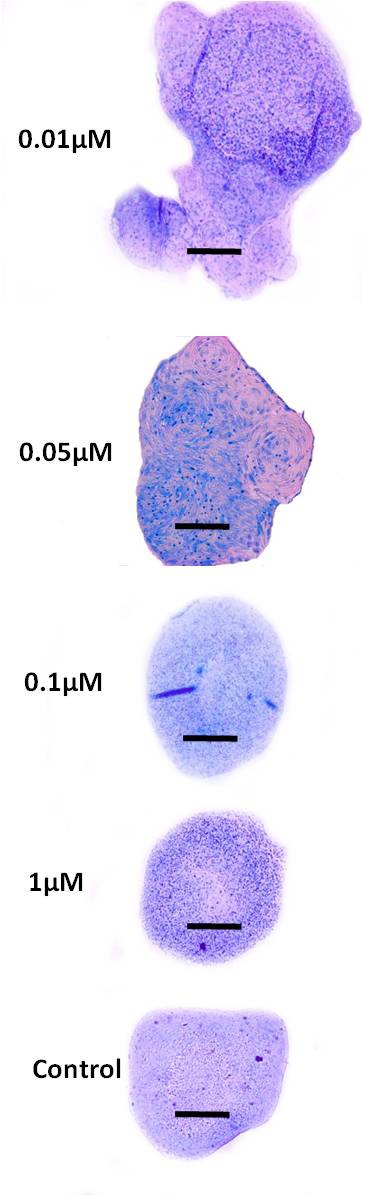
Representative sections from the chondrogenic culture of mouse passaged-3 MSC at day 21. Relatively more metachromatic matrix seemed to be produced at the culture treated with 0.01 µM BIO (Bar=200 µm).

Light microscopic study revealed that a metachromatic matrix was produced in all the BIO-treated chondrogenic cultures as well as the control. Figure 3 indicates representative sections from the middle of the chondrogenic pellets developed in the different chondrogenic cultures. Apparently, the amount and intensity of the metachromatic matrix is higher in the culture treated by 0.01 µM BIO. Metachromasia is the property imparted to the cartilage tissue by glycosaminoglycan-rich proteoglycan such as aggrecan.

*Expression Pattern of Cartilage-Related Molecules*

**Figure 4:**
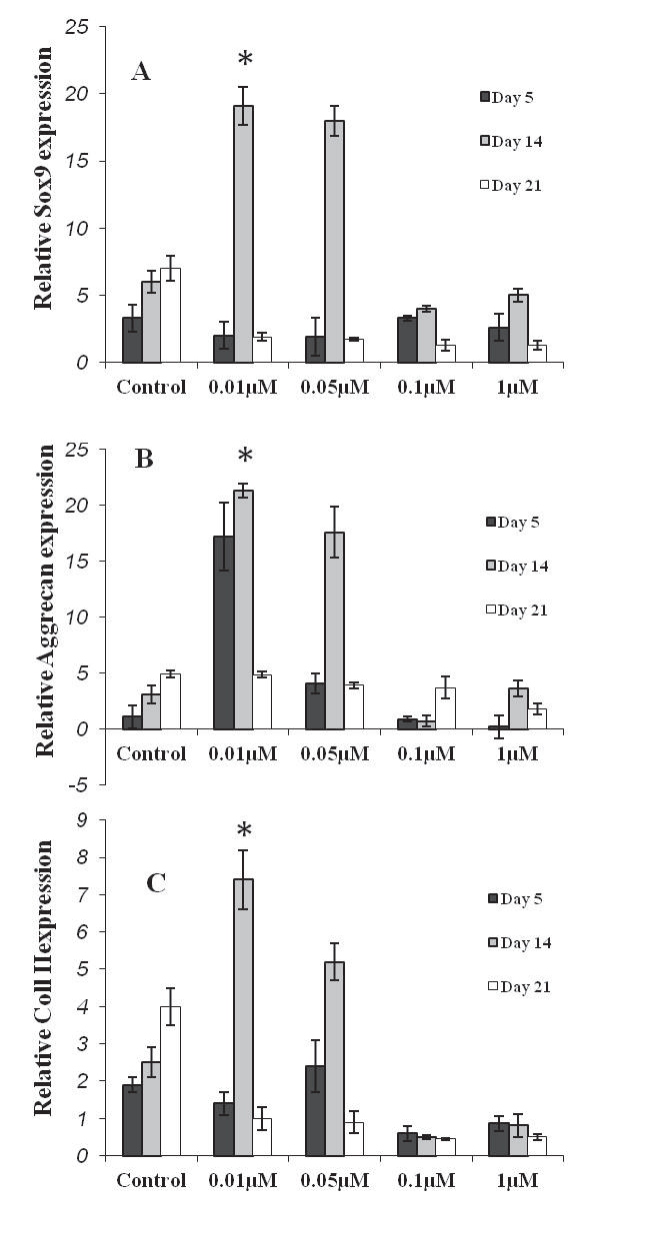
Expression pattern of three cartilage-specific genes. A) Sox 9: This transcription factor upregulated at day 14 of the cultures treated by 0.01 and 0.05 µM BIO (*indicates a P=0.01). In the control group, upregulation of this molecule occurred at day 21, indicating the delayed differentiation of the cells in this group compared to that in 0.01 and 0.05 µM BIO-treated cultures. This pattern of expression occurred regarding both B) aggrecan and C) collagen II expression.

**Sox9: **At day 5, the expression level of Sox9 was low at all the BIO-treated cultures. At day 14, there was a statistically significant increase in the expression level of Sox9 in cultures with 0.01 and 0.05 µM BIO (P=0.01). In this regard, the level of expression was higher in the culture with 0.01 than 0.05 µM BIO. At day 21, this cartilage-specific transcription factor was downregulated at all the BIO treated-cultures, so that there was no statistical difference between the groups. In the control group, Sox9 expression reached a peak at day 21 (figure 4A).

**Aggrecan: **At day 5, there was relatively a significant upregulation of aggrecan in cultures with 0.01 µM BIO (P=0.01). In this group, the level of aggrecan expression was further increased at day 14 compared with that in the other groups. At day 21, the expression level of this cartilage-specific gene decreased in all the BIO-treated groups (P=0.05). At this day, there was no significant difference between the cultures (figure 4B). In the control group, aggrecan appeared to be upregulated at day 21.

**Collagen II: **Regarding collagen expression, a similar pattern as with Sox 9 was observed in the studied cultures (figure 4C).

*Expression Pattern of Wnt-Related Molecules*

**Figure 5:**
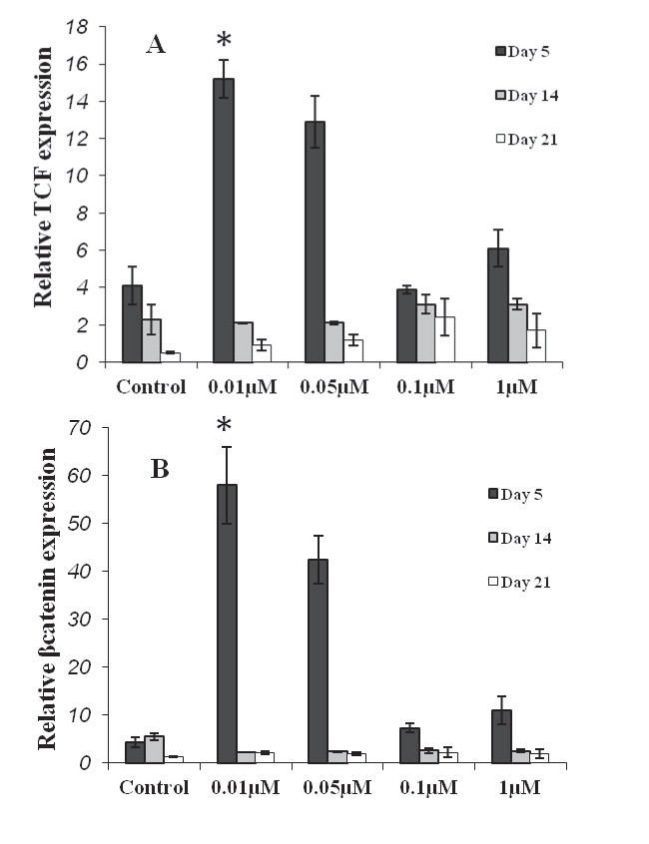
Relative expression of the Wnt-related molecules. A) TCF as the key transcription factor in the Wnt-pathway upregulated at day 5 in all the studied culture. In this term, the culture with 0.01 µM BIO had a significant difference compared to the others (*indicates a P=0.01). The level of TCF expression was decreased as culture progressed. B) A similar pattern of expression occurred regarding beta catenin.

At day 5, TCF (T-cell factor), a key molecule of the Wnt signaling pathway, was upregulated in all the studied cultures. In this regard, the upregulation level was higher in chondrogenic culture with 0.01 µM BIO (P=0.01). This marker of the Wnt pathway was progressively downregulated towards the end of the cultivation period in all the studied cultures (figure 5A). A similar expression pattern was observed regarding beta-catenin expression, the other key molecule of the Wnt signaling pathway, in the BIO-treated cultures (figure 5B).

## Discussion

Cartilage defects exert a major challenges in the field of orthopedic surgery since the tissue possesses a limited repair capacity owing to its avascular nature. On the other hand, MSCs possess a great promise to promote the regeneration in hyaline cartilage defects. According to one strategy, in the cell-based treatment of tissue defects, cells must be fully differentiated into the desired cell prior to transplantation. Therefore, in the present study, an attempt was made to evaluate the enhancing effects of BIO on the chondrogenic differentiation of marrow MSCs. Our results indicated that the presence of BIO in MSC chondrogenic culture accelerates the differentiation. In addition, supplementation of culture, in particular with 0.01 µM BIO, enhanced differentiation. These data would be of help to those involved in functional cartilage engineering, the ultimate goal of which is to elaborate appropriate cartilage constructs for transplantation into articular cartilage defects.^29^^-^^31^

In the present study, the selection of BIO concentration range was, performed according to the previous reports in which cell proliferation cultures were treated by BIO. Sato et al.^28^ reported that the presence of BIO at concentrations less than 1 µM enhanced murine embryonic stem cell proliferation. Similarly, Sinha et al.^22^ found that the presence of BIO at 0.01, 0.05, and 0.1 µM in culture could increase the proliferation of murine kidney epithelial cells. These reports indicated the positive effect of BIO at the mentioned concentrations on the cell viability and proliferation, meaning that the used dosages had no cytotoxic effects. Using similar BIO concentrations, here, we reported that BIO at 0.01 µM could accelerate and enhance the cartilage differentiation of marrow MSCs. These effects of BIO are easily conceivable considering that it belongs to the category of organic compounds referred to as small molecules which are of low molecular weight capable of binding with high affinity to biopolymers such as proteins, nucleic acids, and polysaccharides altering their activity or function. The most important advantage of small molecules is that they can rapidly diffuse across cell membranes, reach intracellular sites of action, and specifically target the signaling pathway.^32^ Furthermore, we found that the treatment of culture with 0.1 and 1 µM Bio could result in the reduced expression of cartilage-specific genes. One logical explanation for this finding would be that 0.1 and 1 µM BIOmight be toxic on MSC culture. This is not in agreement with the previous findings indicating the promoting effects of 0.1 and 1 µM BIO on the viability of cells.^22^^,^^28^ The other explanation could be admitting that these dosages of BIO were not enough to create chondrogenic effects on the MSC cultures. This issue, however, needs further investigations.

According to our findings, cartilage-specific genes, including Sox9, aggrecan, and collagen II were in their maximum expression levels at day 14 of the cultures treated with BIO (in particular in 0.01 µM), whereas the expression level of these genes reached a maximum at day 21 of the control culture. This implies that BIO accelerates the cartilage differentiation of MSCs. Having MSCs give rise to chondrocytic cells in the short term could be of crucial importance regarding their application in regenerative medicine in two ways: firstly it shortens the waiting time for the patient to undergo transplantation and secondly it decreases the time of the culture period hence diminishing the overall cost of cell preparation. 

In the present study, two key molecules of the Wnt signaling pathway, i.e. beta-catenin and TCF, were also quantified during the differentiation period. These two molecules play a crucial role in the Wnt signaling pathway. When the Wnt ligand binds to its receptor and a co-receptor, the APC/Axin/GSK3β destruction complex is inhibited, leading to the stabilization of beta-catenin and its translocation to the nucleus where it interacts with T-cell factor/lymphoid enhancer factor (TCF/LEF) transcription factors. In the absence of signal, TCF/LEF factors bind DNA at Wnt-responsive genes and interact with other factors (e.g. Groucho, histone deacetylase) to repress transcription.^33^ In all the cultures, in the present study, the expression level of these two molecules was significantly high at day 5 of the BIO-treated cultures and then progressively decreased as the culture progressed. This expressional pattern is logical since the activation of the signaling pathway is prior to the expression of tissue-specific genes.

According to the histological section prepared from the pellet at day 21 of culture, the amount of metachromasia seemed to be higher in cultures with 0.01 and 0.05 µM BIO compared to that of the control and cultures with 0.1 and 1 µM BIO. On the other hand, based on RT PCR, at day 21, the amount of aggrecan mRNA, which is responsible for cartilage metachromatic property, was higher in the control group compared to that in the cultures with 0.01 and 0.05 µM BIO. The explanation would be that the aggrecan mRNA in cultures treated with 0.01 and 0.05 µM BIO tended to be expressed into protein at day 21, while in the culture without BIO, it was in the form of mRNA at day 21 (as was detected by the PCR method). The same is true for collagen mRNA.

In this study, we evaluated the outcomes of BIO addition in terms of the upregulation/downregulation of the cartilage-related genes as well as the Wnt-related key molecules during the differentiation period of marrow MSC chondrogenesis. The production of gene mRNA did not equal to its expression into proteins. Our Toluidine blue staining indicated that the cartilage-specific molecule of aggrecan mRNA converted into protein since metachromatic matrix was present in the sections. Regarding the Wnt molecules, however, further investigation needs to be undertaken to reveal their expression into protein. 

## Conclusion

Taken together, BIO in particular at 0.01 µM concentration could accelerate and enhance the cartilage differentiation of mouse marrow-derived MSC in culture. These data would be helpful for those involved in cartilage engineering for the application in hyaline cartilage regeneration, which is deemed problematic in the field of orthopedic surgery. 
